# Improving Female Adolescents’ Knowledge, Emotional Response, and Attitude toward Menarche following Implementation of Menarcheal Preparation Reproductive Health Education

**DOI:** 10.31372/20190402.1041

**Published:** 2019

**Authors:** Setyowati, Mira Rizkia, Titin Ungsianik

**Affiliations:** Faculty of Nursing, Universitas Indonesia, Depok, West Java, Indonesia

**Keywords:** adolescent, attitude, emotional response, knowledge, menarche, health education

## Abstract

Menarche and menstruation are considered taboos and disconcerting by communities, including female adolescents. The Menarcheal Preparation Reproductive Health Education program was designed to prepare female adolescents for menarche. The aim of this study was to identify the influence of reproductive health education on female adolescents’ preparation, knowledge, emotional response, and attitude toward menarche. The research design was a quasi-experimental, pre–post test with control group design. We selected 174 female adolescents by a stratified random sampling technique. The respondents were divided into two groups: control and intervention. The intervention group was provided Menarcheal Preparations Reproductive Health Education program in the form of a booklet, whereas the control group experienced no intervention. Data analyses involved the use of a chi-square test, McNemar test, and logistic regression. The results showed that there were significant differences in terms of knowledge (*p* = .001), emotional responses (*p* = .001), and attitude (*p* = .001) between the groups, as well as before and after intervention in the intervention group. Logistic regression revealed that reproductive education was the most influencing factor among female adolescents’ knowledge (OR = 45.1; 95% CI: 13.8–148.1), emotional responses (OR = 12.7; 95% CI: 5.6–28.5), and attitude (OR = 12.4; 95% CI: 5.8–26.6) toward menarche. Therefore, this study supports a recommendation of using Reproductive Health Education Related to Menarcheal Preparation in schools and community settings to prepare female adolescents for menarche.

## Introduction

Women’s reproductive health, including adolescent females, is one of the focuses of Sustainable Development Goals (SDGs) and it is one of the goals of WHO to improve equality in health, reduce health risks, and promote healhty lifestyles and setting (WHO, 2014). One major problem is the ill preparedness of females for their first period or menarche (Marván & Alcalá-Herrera, 2014). In 2014 there are 24 million adolescent females in Indonesia who have not yet experienced menarche, which occurs at approximately 13 years of age, but studies suggest that many are not yet prepared for this change in their lives because 25% did not want to discuss menstruation, whereas 17% were unaware that menstruation occurs at the beginning of puberty (Burnet Institute, SurveyMETER, & WaterAid Australia, 2015). The menstrual period and menarche are physiological conditions, but the psychological response of females, including negative feelings, are influenced by the information they receive (Tesfaye, 2017). A study by Tesfaye (2017) reported that only 22.7% of females received appropriate information about menstruation, followed by 22.1% receiving sufficient information, 32% receiving inadequate information, and 23.7% receiving entirely inadequate information. According to the information from the Indonesian Ministry of Health (2015), 57.6% of females use their peers as information sources and for discussions regarding reproduction health, 42.1% use their mothers, 45.1% their teachers, and only 13.7% use formal health providers (Kementerian, 2015).

The inappropriateness of the information sources could influence the emotional responses and attitudes as well as the behavior of females during menarche and menstruation (Chandra-Mouli & Patel, 2017). This also feeds the feeling that menstruation is a taboo and an embarrassment that should not be discussed and instead, kept secret (Jackson & Falmagne, 2013). As a result, at the onset of menstruation, females may exhibit negative emotion responses, attitudes, and behaviors (Tang, Yeung, & Lee, 2004), especially in the communities in risk areas such as villages or prostitute localization (Dovis, Setyowati, & Kurniawati, 2017).

Embarrassment and anxiousness are experienced by females at menarche, and they will avoid going out in public when they are on menarche (Behera, Sivakami, & Behera, 2015). A study by Lahme, Stern, and Cooper (2016) described discriminatory behavior in schools, especially by boys, during situations such as stained uniforms that are perceived to be embarrassing, leading to trauma that will influence their willingness to go to the school and so disturb their education. This will lead to increased school absences, including for examinations, and subsequent poor academic performance (Lahme et al., 2016). Delivering education on reproductive health to adolescents is not adequate yet in developing countries (Sommer, Figueroa, Kwauk, Jones, & Fyles, 2017). Some studies have reported that inconducive school environments cannot fulfill adolescents’ needs during menstruation and that health education that provides emotional supports and not fragmented information is required (Chandra-Mouli & Patel, 2017; Sommer et al., 2017).

Current reproductive health education is still general but not sufficiently specific, for example, the research by Djuwitaningsih and Setyowati (2017) about the development of an interactive health education model based on the Djuwita application for adolescent girls according to their needs. But the information about menarche and menstruation was limited, it only provided general informations, for example, the female and male reproductive organ and prevention of pregnancy (Djuwitaningsih & Setyowati, 2017). Another study provided information on menstrual hygiene only (Chandra-Mouli & Patel, 2017) regarding mapping the knowlegde and understanding of menarche, menstrual hygiene, and menstrual health among adolescent girls. The last study by Rajkumari (2015) focused on knowledge regarding menstrual hygiene among adolescent school girls. Therefore, this study assessed a very specific health education program to prepare females to face menarche by increasing their knowledge of menstruation and their readiness to face the menarche. Being prepared physically and emotionally should lead to more positive attitudes and behavior with respect to reproductive health.

## Methodology

The design of study was a quasi-experimental pre–post test with a control group design. The 174 participants were chosen by stratified random sampling. Eighty-seven respondents were assigned to an intervention group and another 87 to a control group. The inclusion criteria were females who studied in the Madrasah Ibtidayah, were aged 9–12 years old, had not yet experienced menarche, were of Aceh ethnicity, and understood and spoke Indonesian.

Three questionnaires were used during data collection: “Measurement of Knowledge about Menstruation and Puberty,” which was developed by Susan M. Moore (1995) and has been modified in India (Singh, Singh, Arora, & Sen, 2006). It consists of 14 questions, numbers 1–6 in the form of multiple choise with each of the 3 alternative answer questions, and 7–14 with a choice of right or wrong answers. Each item has a value of 1 for the correct answer, and 0 for the wrong answer with a total value of 0–1, mean = 7, good knowledge ≥7 and poor <7, the reability .886. The second questionnaire “Emotional Responses,” developed by Chrisler and Zittel (1998) in Logan (1980) in Tesfaye (2017), grouped answers into positive or negative responses. The positive emotional response include feeling proud, happy, more feminine, and more mature. While negative responses such as shame, fear of worry, pain, and surprise consists of 13 questions with 1–4 Likert scale, with a value of 1 = not at all, 4 = very strong, mean = 33, positive ≥33 and negative <3, the reability .86. The third questionnaire was “Adolescent Menstrual Attitude Questionnaire,” developed by Morse and Kieren (1993) and Davis, Yarber, Bauserman, Scheer, and Davis (1998). It consists of 13 statements divided into 5 positive and 8 negative statements. Assessment using the Likert scale 1–5 with answer 1 is very unsure and 5 is very confident, mean = 36, positive ≥36 and negative <36, the reability .902. The Pearson Product Moment validity test was used for each questionnaires, and it was declared valid with a value ≥.3 for all question items.

Data collection was conducted in May 2018 in the Aceh Besar district. The intervention was a booklet containing health education on preparation for menarche, including information of the reproductive organ, physical changes during adolescence, problems during menstruation, how to deal with such problems, and menstrual hygiene. No intervention was given to the control group, but the booklet was provided to them after completion of the study.

The statistical analysis utilized univariate and bivariate McNemar and chi-square tests. The significance level is <.05, and the logistic regression with backward method.

Ethical approval was provided by the Ethical Committee of the Faculty of Nursing Universitas Indonesia (approval number: 199/UN2.F12.D/HKP.02.04/2018). This study also obtained permission from the Faculty of Nursing Universitas Indonesia, the region district office of Aceh Besar, and from the head of the school. All respondents provided informed consent after being provided information on the research, and parental permission from parents was also obtained.

## Results

There were no significant differences in the characteristics of the respondents in both groups or both groups were homogenous (*p* > .05; Table 1).

**Table 1:** Frequency Distribution and Homogeneity Testing of Respondents in the Intervention and Control Groups (n = 174)

	Intervention group			Control group			
Characteristic	*N*	%		*N*	%		*p*
**Age**							
Pre-adolescent	11	(12.6)		5	(5.7)		.229
Early adolescent	76	(87.4)		82	(94.3)		
**Living with their mother**							
Yes	86	(100)		84	(96.5)		1.000
No	1	(1.14)		3	(3.4)		
**Has older sister**							
Yes	47	(54)		48	(55.2)		.266
No	40	(46)		39	(44.9)		

There were significant differences in knowledge (*p* = .001), emotional response (*p* = .001), and attitudes (*p* = .001) of the females before and after intervention with Reproductive Health Education Menarche Preparation (RHE-MP) (Table 2).

**Table 2:** Differences in Knowledge, Emotional Response, and Attitudes of Females in the Intervention Group Before and After Intervention with RHE-MP (n = 87)

	Before			After			
Variable	*N*	%		*N*	%		*p*
Knowledge							
Good	51	(58.6)		79	(90.8)		.001
Poor	36	(41.4)		8	(9.2)		
Emotional response							
Positive	45	(51.7)		73	(83.9)		.001
Negative	42	(48.3)		14	(16.1)		
Attitudes							
Positive	39	(44.8)		70	(80.5)		.001
Negative	48	(55.2)		17	(19.5)		

There was also significant difference in knowledge (*p* = .001), emotional response (*p* = .001) and attitudes (*p* = .001) in the females regarding menarche between respondents in the intervention and control groups after the implementation of RHE-MP (Table 3).

**Table 3:** Differences in Knowledge, Emotional Response, and Attitudes of Females in the Intervention and Control Groups After Intervention with RHE-MP (n = 174)

	Intervention group			Control group			
Variable	*N*	%		*N*	%		*p*
**Knowledge**							
Good	79	(90.8)		15	(17.2)		.001
Poor	8	(9.2)		72	(82.8)		
**Emotional response**							
Positive	73	(83.9)		29	(33.3)		.001
Negative	14	(16.1)		58	(66.7)		
**Attitudes**							
Positive	70	(80.5)		23	(26.4)		.001
Negative	17	(19 5)		64	(73.6)		

The most dominant factor influencing knowledge of menarche was RHE-MP, which exhibited a positive relationship (OR = 45.1; 95% CI: 13.8–148.1), indicating that females who received RHE-MP had a 45-times higher change of possessing accurate knowledge about menarche than uneducated females (Table 4).

**Table 4:** The Logistic Regression for Female Adolescents' Knowlegde

						(CI 95%)	
Step	Variable	B	Wald	p	OR	Lower	Upper
1	RHE-MP education	3.925	37.709	.001	50.646	14.471	177.249
	Economic state	.791	1.660	.198	2.205	.662	7.345
	Source of information	−3.155	26.483	.001	.043	.013	.142
	Mother and daughter communi-cation	.460	.641	.423	1.584	0.514	4.886
	Constant	2.685	8.461	.004	14.664		
2	RHE-MP education	3.859	37.887	.001	47.414	13.876	162.014
	Economic state	0.880	2.162	.141	2.410	.746	7.788
	Source of information	−3.224	27.848	.001	.040	.012	.132
	Constant	2.973	12.066	.001	19.547		
3	R-PHP education	3.810	39.486	.001	45.141	13.756	148.129
	Source of Information	−3.157	28.166	.001	0.043	0.013	0.137
	Constant	3.154	14.326	.001	23.430		

The most dominant factor that influenced emotional responses to menarche was also RHE-MP (OR = 12.7; 95% CI: 5.6–28.5). Females who received RHE-MP have a 12-times better chance of a positive emotional response about menarche than those who did not (Table 5).

**Table 5:** The Logistic Regression for Female Adolescents’ Emotional Responses

						(CI 95%)	
Step	Variable	B	Wald	p	OR	Lower	Upper
1	RHE-MP education	2.598	31.151	.001	13.433	5.395	33.448
	Economic state	.910	5.043	.025	2.484	1.123	5.494
	Source of information	.121	.079	.778	1.129	.486	2.618
	Mother and daughter communication	1.336	10.512	.001	3.802	1.696	8.524
	Constant	−1.878	4.856	.028	.153		
2	RHE-MP education	2.539	37.630	.001	12.668	5.629	28.513
	Economic state	.910	5.040	.025	2.485	1.123	5.503
	Mother and daughter communication	1.318	10.494	.001	3.736	1.683	8.294
	Constant	−1.662	21.265	.001	.190		

Finally the most dominant factor influencing the females’ attitudes was also RHE-MP (OR = 12.4; 95% CI: 5.8–26.6), indicating that RHE-MP increased the chances of positive attitudes to menarche 12-times (Table 6).

**Table 6:** The Logistic Regression for Female Adolescents’ Attitudes

						(CI 95%)	
Step	Variable	B	Wald	p	OR	Lower	Upper
1	RHE-MP education	2.642	33.884	.001	14.040	5.768	34.174
	Economic state	.962	5.956	.015	2.616	1.208	5.662
	Source of Information	.248	0.321	.571	1.281	.544	3.019
	Mother and daughter communication	.870	4.786	.029	2.386	1.095	5.200
	Constant	−2.235	6.525	.011	.107		
2	RHE-MP education	2.517	41.588	.001	12.385	5.764	26.611
	Economic state	.959	5.913	.015	2.610	1.205	5.656	
	Mother and daughter communication	.832	4.515	.034	2.298	1.067	4.950
	Constant	−1.787	24.997	.001	.167		

## Discussion

The improvement in knowledge about menarche among the females after intervention with RHE-MP in this study was consistent with research by Rajkumari (2015) that reported improvement in knowledge, positive behavior, and positive thinking in female adolescents after delivery of reproductive health education (Rajkumari, 2015). Another study by Griebler, Rojatz, Simovska, and Forster (2017) showed that health education improved the female adolescent cognitive aspect (Griebler et al., 2017). Djuwitaningsih and Setyowati (2017) also reported a similar result upon increasing knowledge of reproductive health among female adolescents using reproductive health education via an internet application (Djuwitaningsih & Setyowati, 2017).

One of the specific characteristics of the respondents in this study was that most of them lived with their mother and had an older sister. Thus, they tended to have better knowledge regarding reproductive health due to good interactions between mother and child (Obono, 2012; Sommer et al., 2017). Other studies reported that majority of females had obtained information about menstruation from their mother just after they got menarche, which may have resulted in the girls undergoing menarche with anxiety and a lack of satisfaction (Al Omari, Razeq, & Fooladi, 2016; Panda & Sehgal, 2009).

Research in Australia and Canada about menarche and menstruation in immigrant populations reported that most of the respondents experienced menarche as a taboo and embarrassing event, and so did not want to talk about it (Hawkey, Ussher, Perz, & Metusela, 2017).

Health education implemented among pre-menarche females aged 9–12 is an appropriate technique because it can prepare them for the onset of puberty. Research in India agrees that reproductive health education should be given as early as possible in the early stages of adolescence (Chothe et al., 2014; Unicef, 2014). Schools are appropriate places where these females can be provided with adequate information and where they can study the information in a conducive atmosphere (Burnet Institute et al., 2015). Schools also need to provide facilities needed by females during menstruation, for example, a clean, functional toilet with sufficient running water (Boosey, Prestwich, & Deave, 2014). Improving the females’ knowledge will in turn improve their behavior and promote good reproductive health by teaching good personal hygiene, leading to the prevention of genital and urinary tract infections (Anand, Singh, & Unisa, 2015; Rajkumari, 2015). This is consistent with study findings in Africa, which found that health education on menstrual hygiene resulted in more effective integration of knowledge and skills into daily living (Boosey et al., 2014).

This study also found that there was an influence of RHE-MP implementation on emotional responses and attitudes among the respondents. This is consistent with a study by Ruspawan and Rosilawati (2011) of female adolescents in junior high school in Bali, which showed that reproductive health education had a positive impact on psychological responses, for example, the fear, sadness, embarrassment, and anger among adolescent females upon menarche, with 60% having negative feelings prior to health education and 100% having positive feeling afterwards (Ruspawan & Rosilawati, 2011).

Menarche is a psychological life situation that is unforgettable by all females, but the emotional responses to it differ with culture (Karakoci, Bingol, & Ocakci, 2014; Panda & Sehgal, 2009; Yeung, Tang, & Lee, 2005).

It has been reported that a majority of pre-pubescent females have emotional expectations that reflect negatively toward menarche (Tesfaye, 2017). Another research showed that females in America face menarche with either negative or positive emotions, although the negative expectations were more dominant (Tesfaye, 2017). However, if they were first prepared with appropriate information coupled with adequate support, they responded more positively toward menarche and menstruation (Chang, Hayter, & Wu, 2010). This intervention, of course, also improved the attitudes of the females toward menarche in this and other studies (Erbil, Felek, & Karakaşlı, 2015).

Negative behaviors of female adolescents toward menarche include escaping to toilets (4.9%) and not knowing what to do except buying the pads (15.4%). Positive attitudes included informing their mother (50.6%) and older sister (7.3%) which was also found in the other studies (Karakoci et al., 2014; Wulandari & Ungsianik, 2013). A WHO survey (2010) found that in the early stages of adolescence, between 10–12 years old, there was limited understanding about physical growth and development compared with the middle stage of adolescence, so that the habits and attitudes of early stage adolescents toward reproductive health, including menarche and menstruation, were poorer (WHO, 2010).

Use of social media can also aid in improving the attitudes of adolescents, and this was more the favorite and more informative method that influenced the attitudes of adolescents (Djuwitaningsih & Setyowati, 2017). Information via several channels need to be tailored to the respondents’ ages and to the value system of the community (Bartholomew et al., 2016; Skoog, Stattin, Ruiselova, & Özdemir, 2013), and is also supplemented with social support from the family, community, or environment so that the adolescents can maximally improve their health status. Because villagers are more socially cohesive than residents of cities and urban areas, people in the urban or city area are more familiar about reproductive health problems (Panda & Sehgal, 2009). The most important factor in improving positive attitudes is how to maintain healthy behavior and protect reproductive health (Dovis et al., 2017). Further research on a broader population that incorporates the effect of culture would also be desirable so that the results could be more generalized.

One limitation of this study is the sampling methodology, which was not randomized, but it nevertheless produced data with important implications. Another limitation include the presence of other female members in the household such as aunts, grandmother, or cousins who may influence and affect female adolescents’ knowlegde besides living with a mother and having an older sister.

## Conclusion

The results from this study show that the reproductive health education with respect to the preparation for menarche (RHE-MP) effectively improves knowledge, emotional responses, and attitudes of the female adolescents toward menarche. This health education is therefore appropriate for female adolescents in the early stages of puberty, and nurses can use RHE-MP when providing reproductive health education.

## Acknowledgements

We would like to thank the District Officer of Region in Aceh Besar and the Madrasah Islamic schools who had participated in this study. We would also like to thank the Directorate of Research and Community Services Universitas Indonesia.

## Declaration of Conflicting Interests

The authors declared no potential conflicts of interest with respect to the research, authorship, and/or publication of this article.

## Funding

This work is supported by Hibah PITTA 2018 funded by DRPM Universitas Indonesia (Numb. 1854/UN.R3.1/HKP.05.00/2018). And also the appreciation is given to the district officer of Religion in Aceh Besar and the Madrasah Islamic schools that had participated in this study.
